# Immunological monitoring for prediction of clinical response to antitumor vaccine therapy

**DOI:** 10.18632/oncotarget.25274

**Published:** 2018-05-11

**Authors:** Irina N. Mikhaylova, Irina Zh. Shubina, George Z. Chkadua, Natalia N. Petenko, Lidia F. Morozova, Olga S. Burova, Robert Sh. Beabelashvili, Kermen A. Parsunkova, Natalia V. Balatskaya, Dmitrii K. Chebanov, Vadim I. Pospelov, Valeria V. Nazarova, Anastasia S. Vihrova, Evgeny A. Cheremushkin, Alvina A. Molodyk, Mikhail V. Kiselevsky, Lev V. Demidov

**Affiliations:** ^1^ N.N. Blokhin Russian Cancer Research Center, Moscow, Russia; ^2^ Laboratory of Genetic Engineering, Institute of Experimental Cardiology, Russian Cardiological Research and Production Complex, Moscow, Russia; ^3^ Diagnostic Laboratory Clinical Center “Optimum”, Sochi, Russia; ^4^ Department of Immunology and Virology, Moscow Helmholtz Research Institute of Eye Diseases, Moscow, Russia; ^5^ RUDN University, Moscow, Russia; ^6^ “Genetic Technologies and Analyses”, Moscow, Russia

**Keywords:** cancer vaccine therapy, melanoma, predictive markers, B2-microglobulin

## Abstract

Immunotherapy has shown promising results in a variety of cancers, including melanoma. However, the responses to therapy are usually heterogeneous, and understanding the factors affecting clinical outcome is still not achieved. Here, we show that immunological monitoring of the vaccine therapy for melanoma patients may help to predict the clinical course of the disease.

We studied cytokine profile of cellular Th1 (IL-2, IL-12, IFN-γ) and humoral Th2 (IL-4, IL-10) immune response, vascular endothelial growth factor (VEGFA), transforming growth factor-β 2 (TGF-β 2), S100 protein (S100A1B and S100BB), adhesion molecule CD44 and serum cytokines β2-microglobulin to analyze different peripheral blood mononuclear cell subpopuations of patients treated with dendritic vaccines and/or cyclophosphamide in melanoma patients in the course of adjuvant treatment.

The obtained data indicate predominance of cellular immunity in the first adjuvant group of patients with durable time to progression and shift to humoral with low cellular immunity in patients with short-term period to progression (increased levels of IL-4 and IL- 10). Beta-2 microglobulin was differentially expressed in adjuvant subgroups: its higher levels correlated with shorter progression-free survival and the total follow-up time. Immunoregulatory index was overall higher in patients with disease progression compared to the group of patients with no signs of disease progression.

## INTRODUCTION

Immunotherapeutic approaches are at the forefront of cancer treatment, and it has been accepted that patient's own immune system often provides the best weapon to inactivate malignant cancer cells in the body. However, the responses to such therapy (which includes dendritic cell vaccine, PD-1 inhibitory therapy and others aimed at restoring anti-cancer immune function) are very heterogeneous, and ability to monitor and have prognostic markers for such treatments is necessary. Immunologic monitoring of several kinds of immune therapy in melanoma is the focus of the present research study.

The T-cell effector immune response is realized via polarization of Th1 cells and secretion of IFN-γ, IL-2 and IL-12 cytokines, while the humoral immune response is characterized with the predominance of Th2 cells and cytokines like IL-4 and IL-10. Advanced melanoma patients are known to have Th1/Th2 imbalance and low levels of IFN-γ [1]. IFN-γ is a potent activator of mononuclear cells, it induces and modulates the expression of MHC antigens by macrophages, T-cells and B-cells, and also tumor cell lines [2]. Inhibition of IL-2 causes the accumulation of immunosuppressive substances like gangliosides, which are produced by melanoma cells and inhibit the production of IL-2 by directly damaging the molecules [3, 4]. IL-12 induces Th0-Th1 polarization and Th1-cells secrete IFN-γ. IL-12 family cytokines play the critical role in the differentiation of Th-cells and regulation of immunologic reactivity. Dendritic cells (DC) and macrophages are the major producers of IL-12. There are both positive and negative dynamics of IL-12 during immunotherapy in melanoma patients described earlier [5]. The key role of IL-6 as a negative prognosis factor of overall survival was repeatedly reported [6].

IL-10 plays an important role in the Th2 antitumor immune response [7], with high levels of IL-10 correlating with poor survival [8]. In addition to serum immunosuppressive cytokines like IL-10 and TGF-b2, proangiogenic bFGF and VEGF are elevated in patients with metastatic melanoma. The identified inversion between IL-10 and VEGF levels in melanoma patients requires further investigation [10]. VEGF may inhibit the maturation of dendritic cells before presentation of tumor antigens to T-cells causing malfunction of antitumor immune response [9].

β2-microglobulin, which is part of the light chain of MHC class I molecules that present antigens to CD8+ cytotoxic T-cells and thus are necessary for the immune T-cell recognition, may be considered another predictive marker. While β2-microglobulin is exfoliated from the cell surface, it can be detected in serum and urine. To evade immune surveillance tumor cells lose MHC class I and β2-microglobulin molecules, and early defect of the β2-microglobulin may cause the selection of highly aggressive melanoma cells leading to a total loss of MHC class I molecules. Mutations of β2-microglobulin gene correlate with the loss of MHC class I antigens and melanoma progression [13].

In the present work, we aim to study the levels of these critical molecules in the context of treatment with cancer dendritic cell vaccines. Using our vaccine preparation and administration protocols, we achieve a significant response to the vaccine therapy and by analyzing immunological markers pre- and post-vaccination we identify the markers that may be used for monitoring the therapeutic success and potentially have a predictive value for the clinical outcome.

## RESULTS

### Patient characteristics

The clinical study included 69 patients (Table 1) with stage III-IV melanoma who received treatment at the N.N. Blokhin Russian Cancer Research Center from 1999 to 2002. All patients provided a written informed consent. All patients underwent surgery for metastatic disease before vaccine therapy.

**Table 1:** Distribution of melanoma patients who received vaccine therapy

Arm	Vaccine	Regimen study	n
DC	Dendritic vaccine	Adjuvant	41
Mela	Melavak	Adjuvant	15
DC+Cph	Dendritic vaccine + cyclophosphamide	Adjuvant	13

Prior to vaccine therapy the patients had standard evaluations including physical examination, clinical and biochemical blood tests, chest X-ray, ECG, ultrasound of peripheral lymph nodes, chest and abdominal CT scan, brain MRI. Blood and serum samples were drawn before the start and at the end of vaccine therapy for PBMC immunophenotype evaluation and serum cytokine assays. The reference group included 30 healthy volunteers for immunologic control.

### Il-4 and IL-10 cytokine levels correlate and may predict the response to vaccine therapy in the dendritic vaccine group

**Figure 1:**
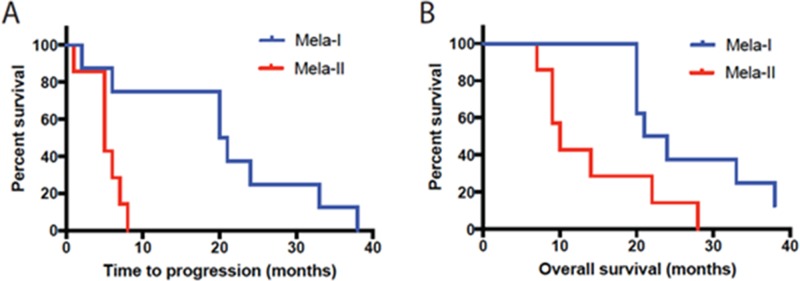
**(A)** Time to progression and **(B)** Overall survival of patients in groups Mela-I and Mela-II (Melavak arm).

First, we analyzed immunologic characteristics such as cytokine levels in the adjuvant setting of the DC arm (dendritic vaccines only). We divided patients into 2 subgroups according to time to progression (TTP): TTP>10 months (subgroup 1), <10 months (subgroup 2), (Table 2, Figure 1A, 1B) and compared these levels with those of healthy volunteers. Th1 cytokines IL-2, IL-12, IFN-γ did not significantly differ between groups 1 and 2, but there was a significant difference as compared with healthy volunteers (p <0.001).

**Table 2:** Time to progression in the adjuvant setting of dendritic vaccine (DC arm)

Group	n	Time to progression, months
**Group 1**	16	46 ± 5
**Group 2**	25	9 ± 1

**Figure 2:**
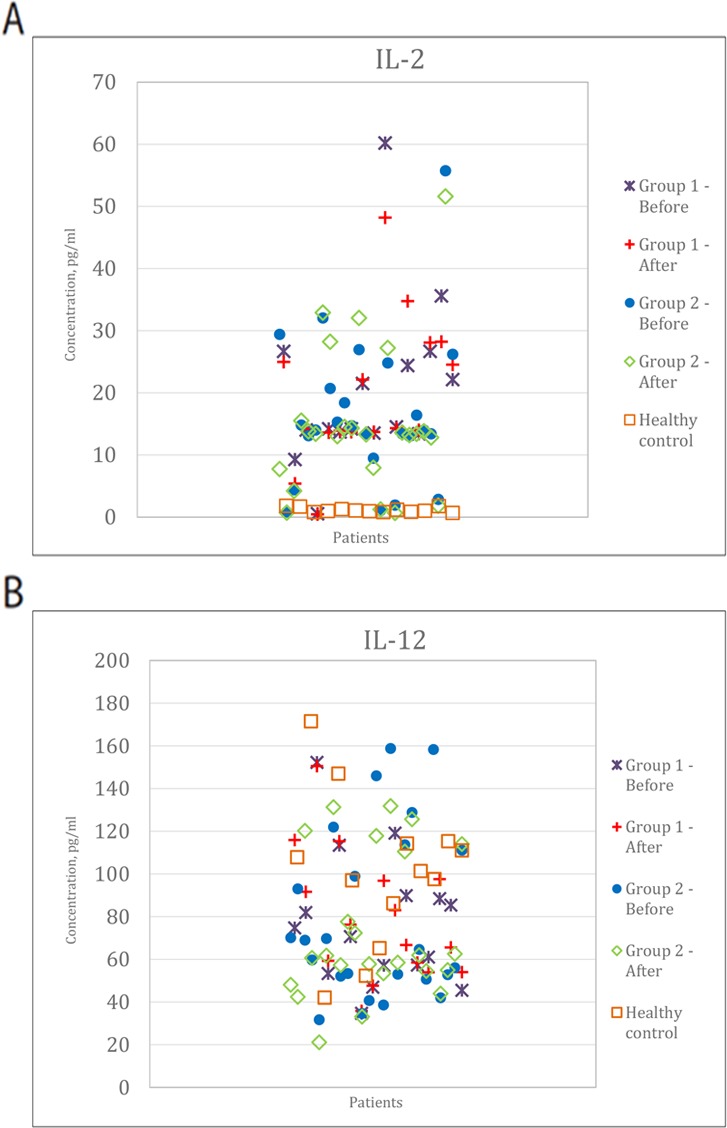
Cytokine profile in two groups of patients compared to healthy control **(A)** Change in IL-2. **(B)** Changes in the IL-12.

The level of IL-4 in group 1 was lower than in group 2 and similar to that of healthy volunteers. In group 2, with shorter time to progression, the level of IL-4 was 3 fold higher as compared to that of healthy volunteers (Figure 2). Interestingly, IL-4 levels did not change during the course of vaccination. IL-10 was 1.2-1.7 fold above the top limit of normal range (ULN), and was lower in group 1 as compared to group 2. IL-10 Levels did not change during vaccination course as well. Thus, the increased levels of IL-4 and IL-10 strongly correlate with the decrease in time to progression in the adjuvant setting of DC vaccine administration. Therefore, IL-4 and IL-10 levels are supposed to be more thoroughly studied as predictive biomarkers for DC vaccine response. It seems reasonable since these cytokines did not significantly change during the course of the treatment.

### S100 levels inversely correlate with response to dendritic cell vaccination

S100 protein is the member of Ca^2+^-binding protein superfamily found in various tissues, and increased serum S100 (ββ) is detected in glioma, melanoma and high-grade neuroblastoma patients [11]. Increased S100 is dependent on melanoma stage and high serum S-100 correlates with poor survival and considered to be a specific marker of tumor progression [12]. We measured serum S100, as well as TGFb2, CD44, VEGFA2 levels in groups 1 and 2 and compared them with these protein levels in healthy volunteers.

Strikingly, S100 was not significantly different in group 1 and healthy volunteers, whereas group 2 patients had significantly higher levels of this biomarker (Table 3, Figure 1B). During vaccination the levels of S100 in group 2 increased even higher. TGFb2, CD44, VEGFA2 did not change during vaccination but were significantly different from those of healthy volunteers (p<0.001).

**Table 3:** Some biomarkers in melanoma patients (DC arm) with different progression patterns in comparison with healthy volunteers

Treatment	Group number	n	Concentration (M±m)							
			S100 ng / L		TGF b2 pg / ml		CD44 ng / ml		VEGF A2 pg / ml	
			before	after	before	after	before	after	before	after
Adjuvant (DC arm)	1	16	55.9 ± 3.9	63.5 ± 6.4	1205.5 ± 96.9	1211.5 ± 104.5	543.0 ± 80.9^*^	500.4 ± 31.3^*^	191.3 ± 50.2^*^	180.2 ± 53.1^*^
	2	25	97.1 ± 9.9^*^	150.1 ± 16.3^*^	1320.7 ± 126.0^*^	1297.9 ± 71.1^*^	531.6 ± 52.6^*^	525.2 ± 51.2^*^	144.8 ± 27.4^*^	132.9 ± 21.9^*^
Healthy volunteers		13	69.4 ± 9.8^*^		1218.6 ± 28.6^*^		347.7 ± 20.5^*^		74.9 ± 25.2	

p <0.05^*^.

### Engineered vaccine Melavak produces durable increase of time to progression and survival in a subgroup of patients

Another vaccination protocol included engineered cell vaccine Melavak producing GM-CSF. In our setting a very significant increase of time to progression and overall survival was achieved in Mela-I subset of patients (over 20 months in subgroup Mela-I compared to 5.2 months in subgroup Mela-II, Fig. 1A and 1B, Table 4). We analyzed serum β2-microglobulin in the group of 15 melanoma patients before and after vaccine therapy with Melavak (Table 4) and compared it with that of healthy volunteers. β2-microglobulin level in healthy volunteers was 2.14±0.88 mg/l, while similar levels of β2-microglobulin were registered in 2 melanoma patients only. The level of β2-microglobulin in all other patients ranged from 4.0 to 7.0 mg/l. Interestingly, Mela-I subset, including 8 patients, demonstrated lower initial β2-microglobulin levels (with the highest values <4.5 mg/l), and β2-microglobulin levels decreased even lower during treatment. Mela-II subset included 7 patients and demonstrated high initial levels of β2-microglobulin, which did not decrease over the course of treatment. It is important to point out that Mel Kor cell line, which was the base for Melavak vaccine, is characterized by the lack of β2-microglobulin and MHC class I molecules.

**Table 4:** Levels of β2-microglobulin before and after adjuvant vaccine therapy

Group number	Level of β2M mg/L		Median time to progression (months)	Median survival (months)
	prior to vaccination	after vaccination		
Mela-I	4.2±1.9	3.2±0.7	20.5	24.1
Mela-II	5.3±1.3	6.4±1.0	5.2	6.3
The control group of volunteers (n=13)	2.14 ± 0.88			

Thus, 8 out of 15 patients with no significant increase of initial β2-microglobulin, had significantly improved time to progression and overall survival (p<0.05). Patients with rapid progression had high initial levels of β2-microglobulin that did not decrease during the course of vaccination. Therefore serum β2-microglobulin level may be a predictive marker for identifying the patients who could have increased time to progression and survival while treated by engineered vaccine Melavak.

### Immunoregulatory Index correlates with response to treatment in DC vaccine+Cyclophosphamide patient group

Patients treated with dendritic vaccine + cyclophosphamide group (DC+CPH, arm3) were subdivided into the following subgroups: arm3A (patients who received 6-11 vaccine injections and their treatment was discontinued due to disease progression) and arm 3B (patients who completed the whole course of immunotherapy (7-17 vaccinations) with no evidence of disease progression at the time of data evaluation).

We evaluated the immune status of melanoma patients who received dendritic vaccine + cyclophosphamide. The immunophenotype analysis included the following subsets: mature T-cells (CD3+), T-helper cells (Th, CD4+), cytotoxic T-cells (CTL, CD3+/CD8+), natural killer cells (NK, CD16+, CD56+), natural killer T-cells (NKT, CD16+/CD3+, CD56+/CD3+), regulatory T-cells (T-reg, CD4+/CD25+).

There were no significant differences of surface antigen expression between groups 3A and 3B (unresponsive and responsive to DC + Cph treatment). Expression of CD3+ varied from 25 to 90% in group A and from 10 to 90% in group B (normal values: 60−80%), CD4+ − from 5 to 65% in group A and from 10 to 65% in group B (normal values: 35 - 50%). The number of cells expressing CD3/CD8 was from 3 to 50% in group A and from 12 to 45% in group B (normal values: 19−40%). Number of NK cells (CD16+ or CD56+) varied from 3 to 28% in group A and from 5 to 90% in group B (normal values: 5−20%). Subpopulation of cells expressing markers of NK and T cells (CD16+/CD3+, CD56+/CD3+) ranged from 3 to 35% (normal values: 0−4%). However, we noticed significant fluctuations of the expression rate of these markers when analyzing that in the course of treatment.

**Figure 3:**
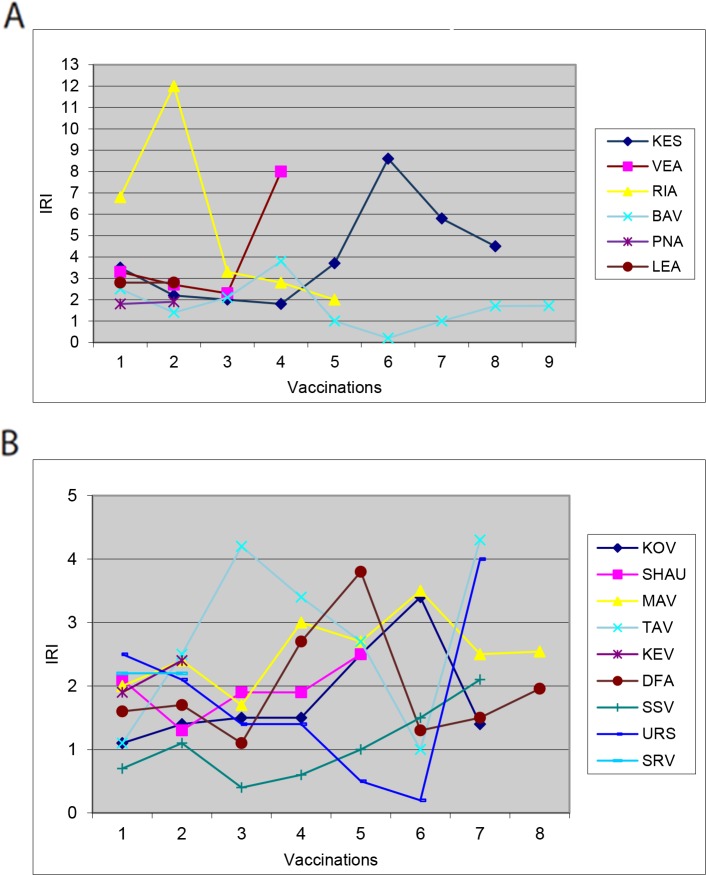
Immunoregulatory index profile in two groups of pati1ents **(A)** Change in IRI, Group A. **(B)** Changes in the IRI Group B.

Immunoregulatory index (IRI) was calculated for both groups (CD4/CD8 ratio). In group 3A of the DC + Cph arm the index ranged from 1.0 to 12.0, while in group 3B it spanned from 1.1 to 4.0 (normal value: from 1.5 to 2.0) (Figure 3). Immunoregulatory index was overall higher in group A (patients with disease progression) than in group B (patients with no signs of disease progression). It is well established for infectious diseases that T-helper cell generation increases during infection, and IRI reduction is associated with an increase of CTLs. Deviations of the immunoregulatory index may indicate any kind of an inadequate immune response [14].

## DISCUSSION

Nowadays, it is widely recognized and the scientific community has strong evidence to support the idea that the immune system can prevent tumor growth or cause tumor rejection and mechanisms of tumor escape from immune surveillance have been intensively studied over the last decades. Yet, we need more data for better understanding of these processes to prevent tumor growth and fight against cancer. Therefore, immune monitoring and early prognostic evaluation may become important tools for effective clinical management and immunotherapy of cancer. We suggest a number of key measurements that may have prognostic value in various immunotherapy approaches, such as specific cytokines and other biomarkers, β2-microglobulin, and IRI, which are available in laboratory tests.

Analysis of T cell immunity and humoral immunity, that is Th1- and Th2-responses, involves evaluation of cytokine levels in relation to the disease stage. It is assumed that immune homeostasis of healthy people is closely related to the balance between Th1/Th2 responses. Th1 response plays the crucial role in antitumor immune reaction and it stimulates activation of CD8+CTLs that have killing potential against proliferating tumor cells. Th1 polarization of T-cells is determined by cytokines such as IL-2, IL-12, IFN-γ and TNF-a [31], while Th2 response is determined by cytokines IL-4, IL-10, IL-5, IL-6.

Analysis of the humoral immune response to vaccination showed that most patients with no detectable tumor (adjuvant treatment) had low IL-4 and IL-10 initial values. The patients with extended time to progression had significantly reduced IL-4 and IL-10 values similar to normal rates and these parameters demonstrated no significant dynamics in the course of vaccination. According to some authors IL-10 plays a dual role in the tumor microenvironment [32]. IL-10 secreted by the tumor maintains chronic inflammation status in the tumor microenvironment and inhibits activation of the adaptive immunity [33]. Another report [34] suggests that IL-10 produced by CD4+T-cells functions as an anti-proliferative cytokine [30].

A lot of researchers have reported about the imbalance of cytokines in patients with melanoma - statistically low levels of IL-2 and IFN-γ and high levels of IL-4, IL-6 and IL-10 that reflects the imbalance between Th1/Th2 immune responses [35]. The prevalence of Th2-type immune response may occur as a result of both humoral immune response and «chronic inflammation». The association of inflammation and tumor progression was recently supported by some reports [36, 37]. A comparative study of immune homeostasis in healthy volunteers with benign nevi and advanced melanoma patients (n=209) showed the predominance of Th2 cytokine polarization in melanoma patients involving VEGF as Th2 mediator. The results suggested that advanced melanoma patients formed the systemic Th2 immune response resembling chronic inflammation with production of VEGF. It differs significantly from the Th1 immune homeostasis that develops in patients with acute inflammation emerging after surgical treatment. Metastatic melanoma with higher serum VEGF level is associated with poor prognosis [38] and VEGF produced by the tumor may cause Th2 immune polarization contributing to tumor progression. Our data indicate the predominance of cellular immunity in the first adjuvant group of patients with increased time to progression and shift to humoral immunity with lower cellular immune reaction in patients with short-term time to progression (increased levels of IL-4 and IL- 10).

Other biomarkers play their role as well. Correlation between increased levels of S100 and low life expectancy is considered a specific marker for tumor progression [34]. Enhanced β2-microglobulin levels were registered in some tumors and leukemia [40]. The elevated level of β2-microglobulin was found in patients with thyroid, breast, liver and kidney cancer. β2-microglobulin levels correlated with the severity of the disease, disease stage and tumor mass. In our study elevated β2-microglobulin was associated with the reduced time to progression and overall survival in patients who received adjuvant immunotherapy while low levels of β2-microglobulin indicated a favorable course of disease. Hofmann et al. showed that β2-microglobulin and some other factors (TNF-alfa, sIL-2R) determined the combined predictive value for recurrence as of 82.9% in the multivariate analysis [41].

Finally, chemo-immunotherapy is regarded as a promising combined approach to cancer treatment. Effectiveness of antitumor immunotherapy still has limited rate, but new data have demonstrated that antitumor immune response may control tumor growth in a number of patients with different types of malignancies [15]. Over the last decade the concept of chemotherapy inhibiting the immune response has been revised. The results of several preclinical studies demonstrated that chemotherapy induced the production of regulatory cytokines and specific signals from dying cells mediated via immunogenic cells [16]. As an example, anthracycline-based chemotherapy can stimulate specific immune response potent of killing residual tumor cells or maintaining the disease in the equilibrium phase [17]. Exposure to other chemotherapy groups such as cyclophosphamide, fludarabine, gemcitabine, oxaliplatin and 5-fluorouracil also results in elimination or temporal inactivation of regulatory T-cells and restoration of effector T-cell functions [18, 19]. One of the functions of the regulatory T-cells is suppressing immune responses *in vivo* and *in vitro* directly or via the production of pro-inflammatory cytokines such as IL-10 and TNF which promotes tumor cell growth [39].

These approaches were studied in clinical trials where metastatic melanoma patients were treated with antitumor vaccine and low doses of cyclophosphamide [20, 21]; or chemotherapy resistant cancer patients with disease progression were treated with metronomic regimen of oral cyclophosphamide in the dose of 100 mg daily for 4 weeks. These therapies resulted in reduction of Treg inhibitory activity and restoration of the proliferative activity of effector T-cells and NK cytotoxicity [22]. Gemcitabine increases the IFN-gamma production by activated T-cells and CD69+ cells in pancreatic cancer patients [23], and augments the cell-mediated immune response in small-cell lung cancer patients (phase I clinical trial) [24]. The study of gemcitabine, GM-CSF and low-dose IL-2 combination for treatment of colon cancer patients showed that this regimen had strong immunologic and antitumor activity associated with immunologic events that strongly resembled those induced by cancer vaccine therapy [20]. Cisplatin-based chemotherapy showed reduction of regulatory T-cells in the peripheral blood of patients with advanced melanoma suggesting immunoregulatory potential of this drug [25].

Therefore, at present there is sufficient evidence to prove the feasibility of combining immunologic treatment with chemotherapy. After destruction of tumor cells dendritic cells engulf cellular debris and present tumor antigens to T-cells that leads to the activation of CD4+ and cross-priming of CD8+ T-cells.

Several mechanisms of immunogenic influence of chemotherapy were discussed by some authors [26, 27]. Obeid et al. suggested that the strongest immunogenic agents were anthracyclines (doxorubicin, idarubicin, mitoxantrone). The studies of chemo-immunotherapy on animal models of colon cancer showed that treatment effectiveness did not correlate with the degree of tumor cell apoptosis induced by anthracyclines or other drugs but with the potential of the drugs to induce rapid mobilization of intracellular chaperone calreticulin (CRT) to the surface of tumor cells before initiation of apoptosis. CRT provides an important signal for dendritic cells to process tumor antigens. CRT (originally called calcium-binding protein with high affinity) is responsible for proper folding and transportation of proteins through the endoplasmic reticulum and regulation of different cellular functions such as migration, phagocytosis of apoptotic cells and cytolysis mediated by T-lymphocytes and NK cells. Surface CRT expression level directly correlates with the potential of dendritic cells and phagocytes for phagocytosis and the potential of normal and tumor cell for adhesion to the extracellular matrix.

In the series of preclinical studies with 5-fluorouracil, cisplatin and gemcitabine it was demonstrated that sub-lethal doses may stimulate the expression of tumor-associated antigens, adhesion molecules and downregulate anti-apoptotic genes thus modifying the phenotype of tumor cells to make them more sensitive to the antigen-specific T-cell mediated killing [28, 29]. The clinical trial of neoadjuvant and adjuvant GVAX vaccine for pancreatic cancer as a single agent or in combination with low-dose cyclophosphamide provided the first example of immune-based therapy converting a “non-immunogenic” tumor into an “immunogenic” by inducing T-cell infiltration [30].

Prognostic serum factors in adjuvant therapy may play an important role in management of patients since conventional methods are not always effective in revealing micrometastases or residual disease. The spectrum of cytokines and factors reported in the current study may be used for the immunological monitoring of the immunotherapy effectiveness or the natural course of metastatic melanoma.

## MATERIALS AND METHODS

### Vaccine preparation

Monocyte-derived dendritic cells were generated from the adherent fraction of patient peripheral blood mononuclear cells (PBMC) by culturing with RPMI 1640 plus 2% human AB serum, recombinant granulocyte-macrophage colony-stimulating factor (GM-CSF, 80 mg/ml) and interleukin-4 (10 ng/ml) for 4 days. On day 4 dendritic cells were loaded with autologous tumor lysate, 2 hours later maturation stimuli (tumor necrosis factor-alfa (TNF-a, 10 ng/ml) and prostaglandin E2 (PGE2, 1 ug/ml) were added to dendritic cells. After 48 hours of incubation mature and loaded dendritic cells were harvested, washed, counted, and aliquoted for cryopreservation in liquid nitrogen for subsequent use. Release criteria were the following: typical DC morphological characteristics (veiled cells), DC phenotype as CD83+ (over 70% of total cell population), CD80+, CD54+, CD86+, HLA-DR+ and viability (over 75% of live cells after cryopreservation).

### Tumor lysate preparation

Tumor lysate was used as a source of tumor antigens and was prepared from patient's tumor tissue after surgery. Surgical material was homogenized, tumor cells were washed from debris into PBS and counted. Cell lysates were generated by 4 freeze-thaw cycles (liquid nitrogen, 37°C water bath). Lysed cells were centrifuged (13 000 g), supernatant was sterile filtrated, aliquoted and stored at −80°C.

### Clinical protocols

We studied three cancer vaccines regimens for melanoma patient's treatment, which were developed at the N.N. Blokhin Russian Cancer Research Center.

1. Dendritic vaccine was based on mature autologous monocyte-derived DCs loaded with autologous tumor lysate of melanoma patients. The treatment consisted of 15 repeated intradermal administrations of the dendritic vaccine with 2 - 4 weeks’ intervals for one year. The dose was 2-5×10^6^ dendritic cells **(41 patients).**

2. Vaccine Melavak was genetically engineered from human melanoma cell line (mel Kor) with stable transfection of GM-CSF gene. The same regimen of the vaccine administration course consisted of 15 injections with 2 - 4 weeks’ intervals for one year. The dose per administrationwas 30×10^6^cells **(15 patients**).

3. Dendritic vaccine + cyclophosphamide. This therapeutic regimen included the same mode of dendritic cell vaccination while patients also received cyclophosphamide (CPH) i.v. in the dose of 300 mg/m^2^, a 2-hour CPH infusion for three days before the 1st, 3d, 5th, 7th, 10th and 12^th^ vaccinations with the aim to reduce regulatory T-cells **(13 patients).**

All clinical protocols were approved by Ministry of Health of the Russian Federation.

### Administration of the vaccines

The vaccines were administered intra-cutaneously in the shoulder, groin or umbilical area (2-10 sites) close to the regional lymph nodes. The vaccines were studied in the adjuvant settings.

### Immunologic biomarker analysis

IL-2, IL-12, IFN-γ and VEGF-A were analyzed in serum with the diagnostic test Bender MedSystems (Austria) based on the ELISA assay. IL-10 was analyzed in serum using a diagnostic test «Protein contour» (Novosibirsk) also based on the ELISA assay.

Transforming growth factor 2 (TGF-β2) was measured in the serum using the diagnostic test DRG Diagnostic (USA), the ELISA analysis. S100 (A1B and BB forms) was analyzed in the serum by the diagnostic test (CanAg Diagnostics, Sweden) with a solid-phase, non-competitive method based on direct “sandwich”-technology. sCD44 adhesion molecules was determined in the serum by the ELISA-based method (Bender MedSystems^®^, Austria).

Serum level of β2-microglobulin (β2M) was analyzed by ELISA using the diagnostic test for quantitative determination (ORG5BM-beta-2-Microglobulin, Germany). The results were measured by spectrophotometry at the wavelength of 450 nm. The data were reported in mg/l.

### Immunophenotyping

Cell immunophenotype was analyzed by FACScan flow cytometry (FACSCanto™ II, Becton Dickinson & Co., USA). We used monoclonal antibodies (mAb) conjugated with fluorochromes FITC, PE or APC (Caltag Laboratories, USA) against corresponding cell surface antigens. Analysis of every 5000 gated events was performed by BD FACSDiva software. Immunophenotiping markers included CD3 (mature T-cells), CD4 (Th-cells), CD3/CD8 (cytotoxic T-cells,CTL); CD16, CD56 (natural killer cells, NK), CD16/CD3, CD56/CD3 (natural killer T-cells, NKT); CD4/CD25 (regulatory T-cells, Tregs,). PBMC subpopulations were evaluated before and after vaccine therapy or at the onset of disease progression (if occurred earlier).

### Statistical analysis

Statistical analysis of data was performed using the software package STATISTICA 9.0. Graphs were made using Microsoft Excel. The groups of patients were compared by Paired Student t-test. Results were considered significant at *p* < 0.05. Survival in groups was shown via Kaplan-Meier method and compared by Log-Rank criteria.
